# Trends in Adolescent Suicide by Method in the US, 1999-2020

**DOI:** 10.1001/jamanetworkopen.2024.4427

**Published:** 2024-03-29

**Authors:** Cameron K. Ormiston, Wayne R. Lawrence, Saanie Sulley, Meredith S. Shiels, Emily A. Haozous, Catherine M. Pichardo, Erica S. Stephens, Aleah L. Thomas, David Adzrago, David R. Williams, Faustine Williams

**Affiliations:** 1 Division of Intramural Research, National Institute on Minority Health and Health Disparities, National Institutes of Health, Bethesda, Maryland; 2 Icahn School of Medicine at Mount Sinai, New York, New York; 3 Division of Cancer Epidemiology and Genetics, National Cancer Institute, National Institutes of Health, Rockville, Maryland; 4 National Healthy Start Association, Washington, DC; 5 Pacific Institute for Research and Evaluation, Albuquerque, New Mexico; 6 Division of Cancer Control and Population Sciences, National Cancer Institute, National Institutes of Health, Rockville, Maryland; 7 Department of Social and Behavioral Sciences, Harvard T. H. Chan School of Public Health, Harvard University, Boston, Massachusetts

## Abstract

**Importance:**

Adolescent suicide in the US is a major public health problem, yet temporal trends in suicide methods by demographics are understudied.

**Objective:**

To examine national trends in suicide mortality by method (firearm, poisoning, hanging and asphyxiation, and all other means) from 1999 to 2020 by demographic characteristics.

**Design, Setting, and Participants:**

This serial cross-sectional study used national death certificate data of adolescent (aged 10-19 years) suicide decedents compiled by the National Center for Health Statistics from January 1, 1999, to December 31, 2020. Data analysis was performed from April 1, 2023, to July 9, 2023.

**Exposures:**

Age, sex, and race and ethnicity.

**Main Outcomes and Measures:**

Trends in age-standardized mortality rates and average annual percent change (AAPC) in rates were estimated by age, sex, and race and ethnicity for each suicide method.

**Results:**

This study assessed data from 47 217 adolescent suicide decedents. From 1999 to 2020, suicide by firearm (AAPC, 1.0; 95% CI, 0.1-1.9), poisoning (AAPC, 2.7; 95% CI, 1.0-4.4), hanging and asphyxiation (AAPC, 2.4; 95% CI, 0.2-4.6), and other means (AAPC, 2.9; 95% CI, 1.2-4.6) increased. Rapidly increasing rates were observed among female adolescents for poisoning (AAPC, 4.5; 95% CI, 2.3-6.7) and hanging and asphyxiation (AAPC, 5.9; 95% CI, 5.0-6.8) suicides. From 2007 to 2020, firearm suicides sharply increased among female (annual percent change [APC], 7.8; 95% CI, 6.0-9.5) and male (APC, 5.3; 95% CI, 4.3-6.3) adolescents. Firearm suicide rates increased among Black adolescents from 2012 to 2020 (APC, 14.5; 95% CI, 9.7-19.5), Asian and Pacific Islander adolescents from 2008 to 2020 (APC, 12.0; 95% CI, 9.7-14.5), American Indian and Alaska Native adolescents from 2014 to 2020 (APC, 10.6; 95% CI, 2.6-19.3), and Hispanic or Latino adolescents from 2011 to 2020 (APC, 10.2; 95% CI, 6.3-13.8). During the study period, Black adolescents had the highest average increase in hanging and asphyxiation suicides (AAPC, 4.2; 95% CI, 3.2-5.2). From 2011 to 2020, poisoning suicide deaths increased (APC, 12.6; 95% CI, 8.5-16.7) among female adolescents.

**Conclusions and Relevance:**

Suicide rates increased across all methods from 1999 to 2020. Differences were noted by sex, age, and race and ethnicity. Increasing suicide rates among racial and ethnic minoritized youth are especially concerning, and effective prevention strategies are urgently needed.

## Key Points

**Question:**

How did US adolescent suicide rates change over time by method (firearm, poisoning, hanging and asphyxiation, and all other means) from 1999 to 2020 by age, sex, and race and ethnicity?

**Findings:**

In this cross-sectional study of 47 217 adolescent suicide decedents, suicide by firearm, poisoning, hanging and asphyxiation, and all other means increased from 1999 to 2020. Differences were noted by age, sex, and race and ethnicity in suicide method patterns.

**Meaning:**

Adolescent suicide is an increasing problem, and implementing effective, tailored prevention strategies is increasingly necessary.

## Teaser

This cross-sectional study of adolescent suicide decedents examines trends in suicide methods in the US from 1999 to 2020 by sex, age, and race and ethnicity.

## Introduction

Suicide remains one of the leading causes of death among adolescents in the US. From 2018 to 2021, suicide was the second leading cause of death for adolescents aged 10 to 14 years and the third leading cause of death among those aged 15 to 19 years.^1^ Suicide is linked to numerous, overlapping factors and paths of marginalization, including mental health conditions, historical and intergenerational trauma, discrimination, neighborhood deprivation, accessibility to lethal means, exposure to violence and abuse, and, more recently, the COVID-19 pandemic.^2,3,4,5,6^ Although older and male adolescents have historically had higher suicide rates than younger and female adolescents, respectively,^7,8^ recent evidence suggests these gaps may be closing as suicide rates are increasing more rapidly for female adolescents than male adolescents.^9,10^ Previous reports identified racial and ethnic differences in adolescent suicide; in recent years, the adolescent suicide rate among Black and Asian and Pacific Islander individuals has increased rapidly, whereas the rate among White individuals has decreased.^11,12,13^ Race and ethnicity are social constructs; hence, racial and ethnic differences in health may be driven by social determinants, environment, and systems of oppression and marginalization that produce disparities in health and longevity.^14^

Historically, firearms have been the leading means of adolescent suicide mortality; however, asphyxiation is becoming increasingly common.^9,15,16^ Other common methods of suicide include poisoning and jumping, and methods may vary by demographic characteristics. For instance, among racial and ethnic minoritized male youths, firearms are becoming an increasingly common method of suicide.^8^

Research on temporal trends of the methods of suicide among adolescents remains poorly understood—particularly by age, sex, and race and ethnicity—despite evidence of suicide becoming a burgeoning public health problem and the method of suicide being significantly associated with mortality.^8,13^ Therefore, elucidation of national trends in adolescent suicide methods by demographic characteristics is urgently needed. This study aimed to comprehensively describe trends in suicide methods (poisoning, firearm, hanging and asphyxiation, and all other means) in the US from 1999 to 2020 among adolescents by sex, age, and race and ethnicity. Findings will highlight disparities in suicide methods and priorities for targeted intervention.

## Methods

Information about demographic characteristics and underlying causes of death were obtained from death certificate data compiled by the National Center for Health Statistics from January 1, 1999, to December 31, 2020. Because this cross-sectional study focused on adolescents, we restricted the study to individuals aged 10 to 19 years as defined by the World Health Organization.^17^ We categorized suicide methods into 4 means: firearm; poisoning; hanging, strangulation, or suffocation (hereafter, hanging and asphyxiation); and all other means (not involving firearm, poisoning, and hanging and asphyxiation [approximately 6.5%]) based on *International Statistical Classification of Diseases and Related Health Problems, Tenth Revision* (*ICD-10*) codes (eTable 1 in Supplement 1). Because the leading means of suicide death during the 1999 to 2020 period for poisoning was drug poisoning (78.7%) and the most frequent method in all other means was jumping (34.1%), we further examined suicide deaths due to these specific means. We stratified population estimates of each means of suicide deaths by year, age, sex, and race and ethnicity. Race and ethnicity were categorized using death certificate data as Hispanic or Latino, non-Hispanic American Indian and Alaska Native, non-Hispanic Asian and Pacific Islander, non-Hispanic Black, and non-Hispanic White. Death rates among American Indian and Alaska Native individuals were restricted to Indian Health Service Purchased/Referred Care Delivery Areas to increase the sensitivity of American Indian and Alaska Native race determination on death certificates. The National Institutes of Health Institutional Review Board waived approval and informed consent because the study used publicly available, deidentified data. This study adhered to the Strengthening the Reporting of Observational Studies in Epidemiology (STROBE) guidelines.^18^

### Statistical Analysis

We calculated age-standardized mortality rates by age group (10-14 and 15-19 years), race and ethnicity, sex, and type of suicide means of death (method) using SEER*Stat software, version 8.4.1 (National Cancer Institute). All rates were age standardized to the US population in 2000 and presented as per 100 000 person-years. We used the Joinpoint Regression Program to calculate the average annual percent change (AAPC) in mortality rates, representing the summary measure from 1999 to 2020, stratified by sex, race and ethnicity, and suicide means of death. Using Joinpoint regression, we also calculated annual percent changes (APCs). In this analysis, we identified calendar years with significant changes in trajectories and calculated the slope in each segment. We used the parametric method to calculate 95% CIs for the trend. *P* values were calculated using the permutation distribution of the test statistic (*P* < .05 was considered statistically significant with a 2-sided test). Data analysis was performed from April 1, 2023, to July 9, 2023.

## Results

**Figure 1.**
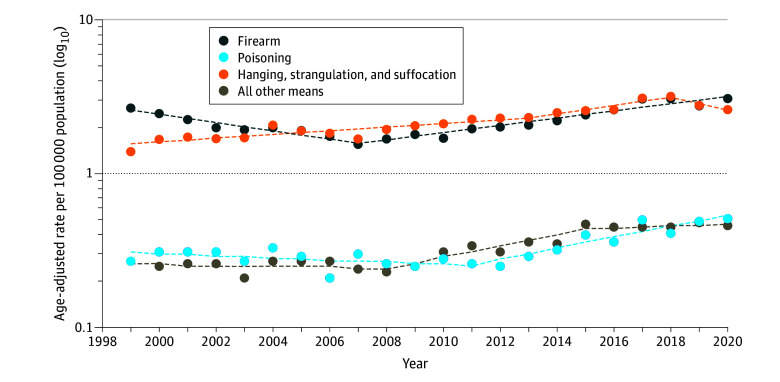
Trends in Age-Standardized Suicide Mortality Rates Among Adolescents by Method, United States, 1999-2020 Circles indicate observed age-adjusted rates; dashed lines, modeled age-adjusted rates.

From 1999 to 2020, 47 217 adolescent suicide deaths were recorded in the US. Firearms (2.2 per 100 000 population) and hanging and asphyxiation (2.2 per 100 000 population) accounted for the highest age-adjusted suicide rates among the total study population (eTable 2 in Supplement 1). On average, death rates for all suicide means increased annually from 1999 to 2020 (Figure 1). Hanging and asphyxiation had the largest absolute increase among male (+1.2 per 100 000 population) and female (+1.3 per 100 000 population) adolescents.

**Figure 2.**
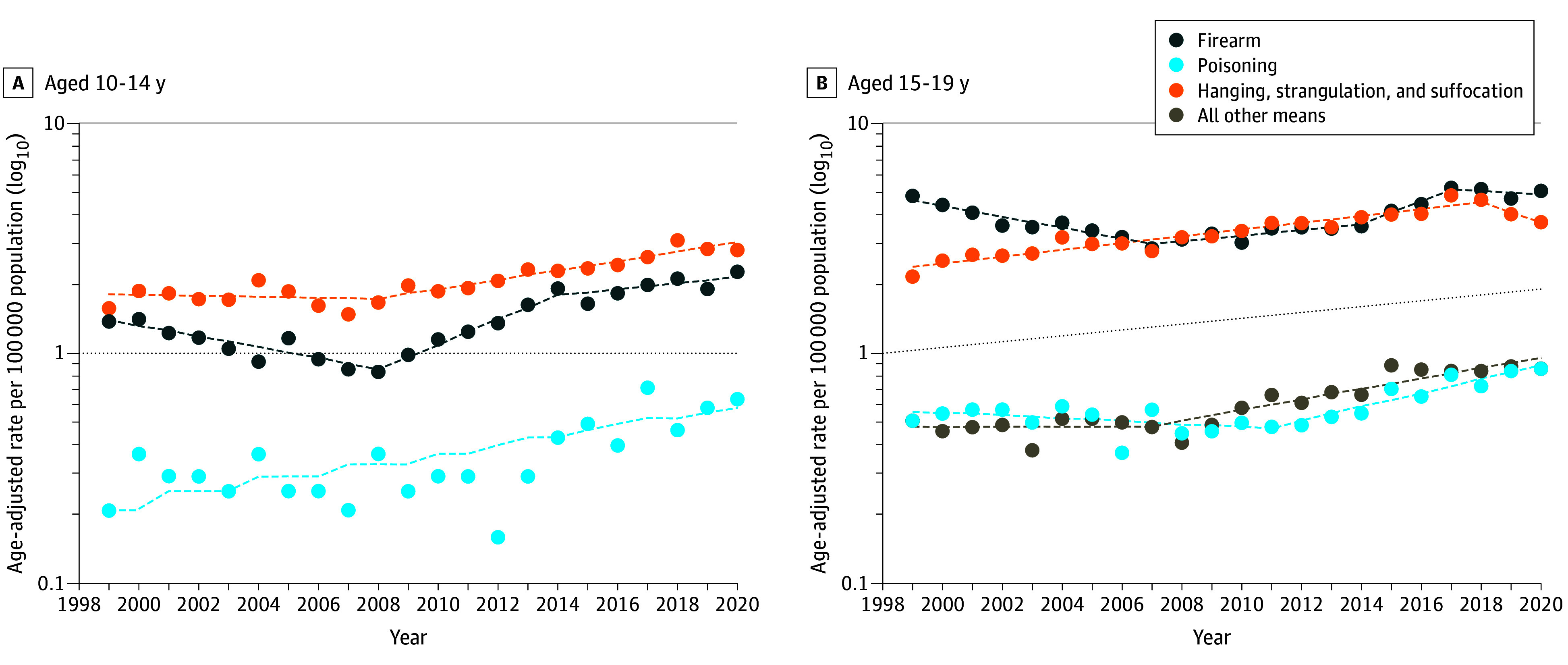
Trends in Age-Standardized Suicide Mortality Rates Among Adolescents by Method and Age Group, United States, 1999-2020 Circles indicate observed age-adjusted rates; dashed lines, modeled age-adjusted rates.

Firearm suicide death rates increased on average 1.0% per year (AAPC, 1.0; 95% CI, 0.1-1.9) (eTable 4 in Supplement 1), with rates decreasing by 6.0% per year (APC, −6.0; 95% CI, −7.9 to −4.2) from 1999 to 2007 then rapidly increasing from 2007 to 2020 (APC, 5.5; 95% CI, 4.6-6.5). American Indian and Alaska Native adolescents had the highest absolute increase in firearm suicide (3.83 per 100 000 population), followed by White (0.69 per 100 000 population), Black (0.67 per 100 000 population), Asian and Pacific Islander (0.64 per 100 000 population), and Hispanic or Latino (0.18 per 100 000 population) individuals. We observed a pronounced increase in firearm suicide death rates among Black adolescents from 2012 to 2020 (APC, 14.5; 95% CI, 9.7-19.5), Asian and Pacific Islander adolescents from 2008 to 2020 (APC, 12.0; 95% CI, 9.7-14.5), American Indian and Alaska Native adolescents from 2014 to 2020 (APC, 10.6; 95% CI, 2.6-19.3), and Hispanic or Latino adolescents from 2011 to 2020 (APC, 10.2; 95% CI, 6.3-13.8) (eTable 5 in Supplement 1). Although rates decreased by 8.0% per year (APC, −8.0; 95% CI, −11.3 to −4.4) for female and by 5.8% per year (APC, −5.8; 95% CI, −7.7 to −3.8) for male adolescents from 1999 to 2007, firearm suicide rates sharply increased from 2007 to 2020 among both groups (APC, 7.8; 95% CI, 6.0-9.5 for female adolescents and APC, 5.3; 95% CI, 4.3-6.3 for male adolescents) (eTable 6 in Supplement 1). When examining by age group, we found an increasing trend in firearm suicides for adolescents aged 10 to 14 years (AAPC, 3.1; 95% CI, 0.3-6.0), with the greatest increase observed from 2008 to 2014 (APC, 20.2; 95% CI, 10.8-30.3) (Figure 2; eTable 7 in Supplement 1).

**Figure 3.**
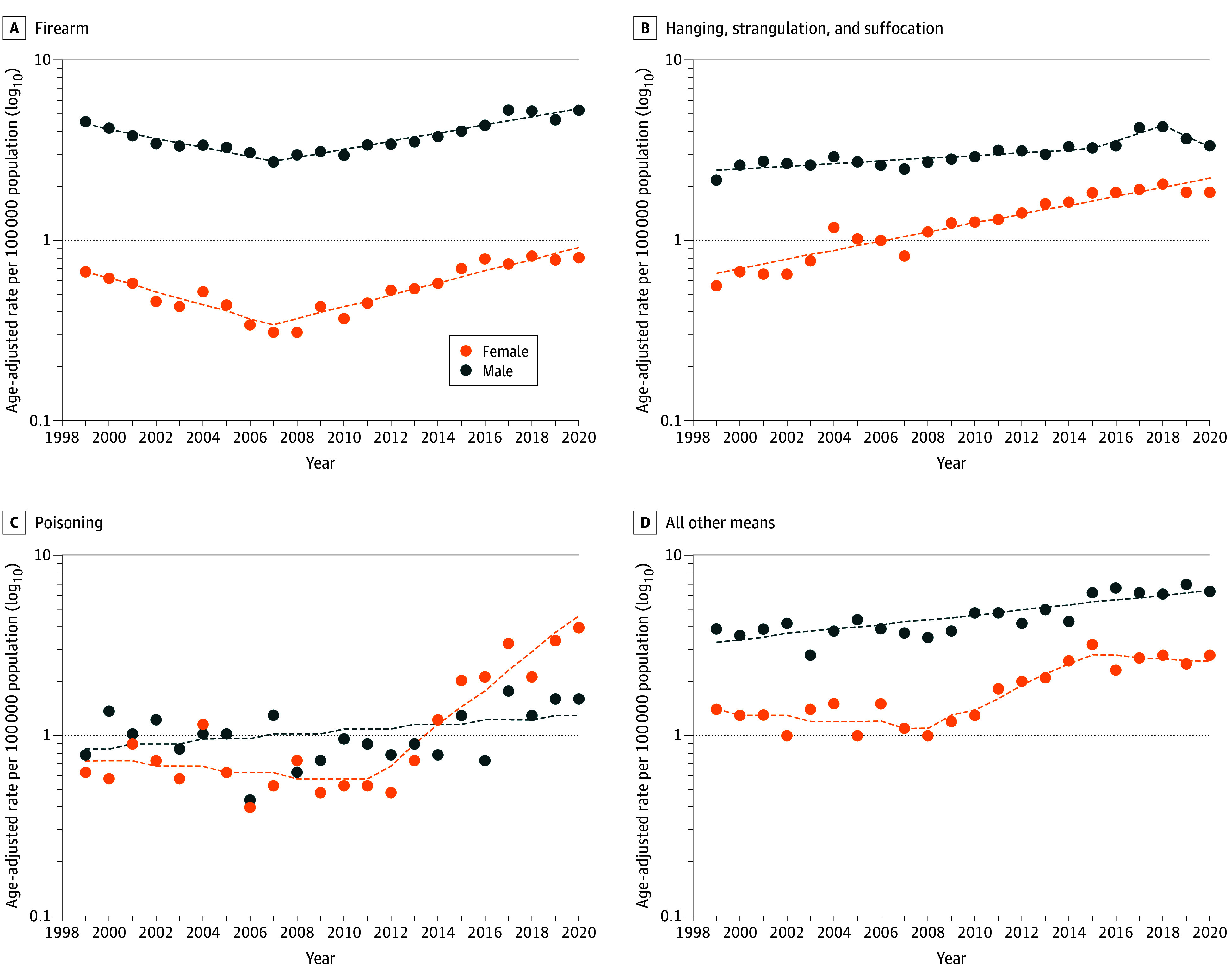
Trends in Age-Standardized Suicide Mortality Rates Among Adolescents by Method and Sex, United States, 1999-2020 Circles indicate observed age-adjusted rates; dashed lines, modeled age-adjusted rates.

Suicide rates caused by poisoning (ie, drug- and non–drug-related poisoning) increased on average by 2.7% (AAPC, 2.7; 95% CI, 1.0-4.4) annually, with the most rapid increase from 2011 to 2020 (APC, 8.6; 95% CI, 5.7-11.7) (eTable 4 in Supplement 1). By sex, average annual increases in suicide rates by poisoning were highest among female adolescents (AAPC, 4.5; 95% CI, 2.3-6.7), with rates accelerating from 2011 to 2020 (APC, 12.6; 95% CI, 8.5-16.7) (Figure 3; eTable 6 in Supplement 1). Drug poisoning suicide death rates exhibited a similar pattern (eTable 9 in Supplement 1). When examining by sex, we found that female adolescents had the largest absolute increase during the study period (0.33 per 100 000 population, 0.12 per 100 000 for male adolescents). Additionally, on average per year, suicide rates for drug poisoning increased more rapidly among female (AAPC, 4.8; 95% CI, 2.5-7.1) than among male (AAPC, 1.4; 95% CI, 0.2-2.6) adolescents. Adolescents aged 15 to 19 years had an average annual 3% increase in suicide drug poisoning death rates (AAPC, 3.0; 95% CI, 1.5-4.5).

Suicide death rates due to hanging and asphyxiation increased annually on average by 2.4% (AAPC, 2.4; 95% CI, 0.2-4.6) (eTable 4 in Supplement 1) and were highest among American Indian and Alaska Native adolescents (12.6 per 100 000 population) (eTable 2 in Supplement 1). The most rapid increase in suicide hanging and asphyxiation death rates was observed among Asian and Pacific Islander adolescents from 2013 to 2018 (APC, 14.7; 95% CI, 4.0-26.5) (eTable 4 in Supplement). On average, female adolescents (AAPC, 5.9; 95% CI, 5.0-6.8) had the highest average annual increase in hanging and asphyxiation suicide rates, and Black adolescents had the highest average increase in hanging and asphyxiation suicides (AAPC, 4.2; 95% CI, 3.2-5.2) (eTable 6 in Supplement 1).

Suicide due to all other means had one of the lowest age-adjusted rates (0.3 per 100 000 population) (eTable 2 in Supplement 1) and increased by 2.9% (AAPC, 2.9; 95% CI, 1.2-4.6) on average per year, with the largest increase observed between 2008 and 2015 (APC, 8.8; 95% CI, 4.8-13.0) (eTable 4 in Supplement 1). Restricting analyses to suicide by jumping showed a decrease in deaths from 1999 to 2008 by 3.7% (APC, −3.7; 95% CI, −6.5 to −0.8) (eTable 8 in Supplement 1). The trajectory changed, however, from 2008 to 2020 because jumping suicides increased by 8.9% (APC, 8.9; 95% CI, 7.4-10.5). Overall, suicides due to jumping increased on average each year for male adolescents (AAPC, 3.2; 95% CI, 1.0-5.4) and individuals aged 15 to 19 years (AAPC, 3.3; 95% CI, 1.6-5.0).

## Discussion

In this nationwide analysis of death certificates, we identified increasing rates of suicide deaths by method, sex, age, and race and ethnicity among US adolescents. Despite variations in suicide rates by method, an overall increasing trend was observed across all demographics. For both female and male adolescents, hanging and asphyxiation suicide rates had the largest absolute increase during the study period. However, the highest average annual increases for poisoning and hanging and asphyxiation suicide rates were observed among female adolescents. These findings highlight the importance of considering sex, age, and race and ethnicity in understanding suicide mortality trends and methods among adolescents.

On average, suicide by firearm increased between 1999 and 2020 and was a leading method. Consistent with prior work, we found that male adolescents had consistently higher rates of firearm suicides than their female counterparts.^8,19,20,21^ This finding is attributed to the higher likelihood of male adolescents residing in gun-owning homes and carrying guns, which are factors strongly associated with suicide risk and mortality.^16,22,23,24,25,26,27^ Notably, although firearm suicide mortality rates appeared to decrease from 1999 to 2007—a trend shown previously^28^—we observed a sharp increase in firearm suicide among both female and male adolescents from 2007 to 2020, which may reflect the surge in new gun owners and youth exposure to gun violence.^29,30,31,32,33^ Additionally, unsafe gun-storing practices persist as a significant nationwide problem and can prove fatal. Adolescent firearm suicides are significantly more likely to occur in homes with unsafe gun storing practices, highlighting an urgent and important area of intervention.^33,34,35,36^

Consistent with prior research,^21,37,38^ American Indian and Alaska Native youth had the highest suicide mortality rates due to firearms from 1999 to 2020. Multiple overlapping factors have been identified as potential contributors to the elevated risk of suicide among the American Indian and Alaska Native community, including intergenerational transmission of historical trauma, high exposure to suicide within American Indian and Alaska Native communities, limited access to mental health and substance use prevention and treatment services, and high rates of interpersonal violence.^13,39,40,41,42^ Additionally, the recent, rapidly accelerating rates of firearm suicide among Black, Hispanic or Latino, and American Indian and Alaska Native adolescents are concerning. These findings, which align with previous studies, underscore an increasing suicide epidemic among racial and ethnic minoritized youths^8,12,21^ and may be influenced by various determinants. For instance, the significant increase in discrimination and hate crimes targeting racial and ethnic minoritized communities during the past decade are recognized contributors to increased suicide risk.^43,44,45,46,47^ In addition, adolescent firearm suicides are more common in neighborhoods with higher concentrations of people living at or below the federal poverty line, which are disproportionately composed of American Indian and Alaska Native, Black, and Hispanic or Latino communities.^48^ Consequently, there is an urgent need to improve access to mental health services and address socioeconomic inequity among the aforementioned communities. It is imperative to address the discrimination, anti-Black violence, and race-based traumatic stress that are increasingly implicated in increasing suicide rates among racial and ethnic minoritized youth^49,50,51,52,53^—particularly Black youth.

Concordant with existing literature,^54,55,56^ mortality rates for poisoning rapidly accelerated between 2011 and 2020 in the current study, especially among female adolescents. Adolescents attempting suicide by poisoning typically choose easily accessed and less lethal drugs.^57^ The ominous change in poisoning deaths may suggest adolescents are finding more lethal means of poisonings, contributing to an increase in deaths by suicide. Differences observed between female and male youths may be due to female adolescents’ higher likelihood to attempt and die by suicide poisoning.^19,58^

Recently observed trends in suicide methods indicate that death by hanging and asphyxiation is rapidly increasing, a pattern consistent with the current study.^8,9,10^ Although the explanation for this trend is unclear, some have pointed to the proliferation of social media use and access to online resources for lethal self-harm, as well as to easier access to means for hanging and asphyxiation among adolescents.^10,55,59,60^ Historically, male adolescents had higher rates of asphyxiation suicide mortality compared with their female peers.^58^ However, previous studies, as well as the current study, observed that rates of asphyxiation suicide among female adolescents are accelerating rapidly, indicating that suicide attempts among female youth may be shifting toward more lethal means.^8,10,28^ This finding is concerning given female adolescents’ higher likelihood of having a suicide plan and their greater rates of suicide ideation and attempts.^58^ Recent upward trends among female youth may be attributable to the increasing influence of social media and its harmful effects on the mental health of female adolescents.^61^

We observed a sharp increase in hanging and asphyxiation suicide deaths among Asian and Pacific Islander youth from 2013 to 2018. Although Asian and Pacific Islander youth have traditionally reported the lowest rate of suicide,^62^ the observed mortality rate increases in highly lethal means (ie, firearms, hanging and asphyxiation) within the last decade underscore suicide as an increasing issue among Asian and Pacific Islander adolescents. Prior research has reported acculturative stress, conflicts of culture reconciliation, overlooked warning signs due to cultural stereotypes, disparities in follow-up care for youth assessed as at risk for suicide, and cultural stigma toward mental health problems as potential contributors to increasing Asian and Pacific Islander youth suicide rates.^63,64^

The increasing adolescent suicide epidemic requires a multifaceted and demographic-specific approach. Solutions must incorporate policy changes and public health initiatives as well as a recognition of the profound and lasting impact of structural racism, systemic inequities, and social determinants of health on suicide. Improving screening and early identification of suicide risk and treatment is paramount; this can be facilitated in primary care visits, through school-based suicide prevention programs, and with awareness campaigns.^55^ These improvements are especially crucial for Black youths, as implicit biases in clinical spaces leading to under-recognition, misdiagnosis, and undertreatment of mental health conditions have been cited as barriers to suicide prevention efforts.^65,66^ For example, Black youth are often mislabeled as having behavioral problems rather than requiring mental health services, which can lead to failures in identifying suicide risk and providing adequate care.^65^

Persistent high rates of firearm suicide among American Indian and Alaska Native adolescents and increasing rates among racial and ethnic minoritized adolescents require urgent attention as firearms and gun culture in the US continue to cause disproportionate harm.^67^ Effective state and tribal child access protection laws as well as the known modifiable methods of reducing access to firearms and improving household gun storage practices can significantly reduce adolescent firearm suicide.^68,69,70^ However, the effects of stricter gun laws are neither equitable nor equal, with the beneficial impact of gun laws on adolescent firearm deaths attenuating as community social vulnerability increases.^71^ This outcome is likely to disproportionately affect racial and ethnic minoritized youths.^71^ Subsequently, focusing on upstream factors to ameliorate structural and neighborhood-level barriers to health (eg, socioeconomic deprivation, residential segregation, systemic racism, underfunded health care systems, and federal infrastructure negligence) is necessary.^71^ Additionally, improving the identification of children at risk of suicide and ensuring parents of at-risk youth are counseled on gun safety and safe storage practices may reduce youth firearm suicide.^32^ For American Indian and Alaska Native youth, interventions that use a protective factor framework, which emphasizes cultural continuity and leverages the strengths of American Indian and Alaska Native communities, hold potential in reducing adolescent suicide.^72^ Restriction strategies to reduce poisoning and drug poisoning suicide, such as disposing of surplus medications, maintaining a home lockbox for all prescription medications, and assessing which medicinal combinations are likely lethal, may be effective.^57,73^ Blister packing opioids and lethal drugs as opposed to storing in bottles has also been suggested to reduce adolescent suicide risk and access to lethal suicide means.^57^ Given the difficulty of restricting access to lethal means outside institutionalized settings for hanging and asphyxiation suicides,^28^ it is crucial to shift our focus toward understanding the underlying reasons for the increase in hanging and asphyxiation suicides, particularly among Asian and Pacific Islander and female youth. By gaining a deeper understanding of the factors that contribute to these trends, we can develop more effective prevention strategies, such as education, screening, and leveraging support systems for at-risk youth.

A strategy that has had promising results among Indigenous (First Nations) youth in Canada suggests that engaging youth in social justice initiatives (advocacy, protest, and empowerment) along with cultural affirmation markedly reduced suicide risks among adolescents.^74^ At the time of the study, First Nations youth in Canada had one of the highest rates of youth suicide in the world. In this seminal study, Chandler and Lalonde^74^ examined youth suicide rates in 196 First Nation communities between 1987 and 1992 and observed that more than half had no youth suicides in the prior 5 years. They identified 5 markers of advocacy, protest, and empowerment (challenging the federal government of Canada over titles to land, the right of self-governance, control over education, access to health care, and police and fire services) and 1 marker of cultural affirmation (establishing buildings where youth participated in cultural activities). They observed that each of these 6 indicators was associated with a lower risk of youth suicide and that there was a dose-response relationship between the number of markers of advocacy, protest, empowerment, and cultural affirmation with the prevalence of suicide. These results were replicated in a later study with subsequent years of data.^75^ More recently, a surveillance system established by the White Mountain Apache Tribe in Arizona reduced adolescent suicide attempts and mortality rates from 2001 to 2014 through an emphasis on culturally driven protective factors and a resilience-focused, culturally based intervention for middle school students.^76^ Data from a randomized clinical trial revealed that increasing social connections by sending brief messages that express care and interest in patients can reduce suicide risk among patients and potentially be effective in reducing suicide risk among American Indian and Alaska Native communities.^77^ The trend data presented here and the availability of potential strategies to reduce youth suicide highlight the urgency of redoubled efforts for sustained initiatives to identify and implement strength-based strategies to reduce suicide risk among US youth.

### Limitations

This study has some limitations, including potential misclassification and errors in suicide mortality on death certificates, particularly in cases of drug poisoning or when suicide intent was unclear. Underreporting or misclassification of suicide deaths also may be high among racial and ethnic minoritized groups due to racial bias in death reporting, potentially obscuring additional disparities.^78,79^ Additionally, we were unable to analyze individual Hispanic or Latino and Asian and Pacific Islander subgroups, which obscures the variability of health outcomes among these racial and ethnic groups.

## Conclusions

Our findings underscore that adolescent suicide in the US is an increasing public health problem as rates for all assessed suicide methods increased from 1999 to 2020. We identified differences in suicide method across sex, age, and race and ethnicity. Although rates increased for all racial and ethnic groups, the increasing rates of firearm, poisoning, and hanging and asphyxiation suicides among American Indian and Alaska Native, Black, and Asian and Pacific Islander youth are particularly concerning. Further research is required to understand the underlying causes of inequities observed in suicide methods and develop effective, tailored strategies to reduce suicide rates among adolescents.

## Notes

Supplement 1. **eTable 1.** External Causes of Death ICD-Codes **eTable 2.** Age-Standardized Mortality Rates Among Adolescents by Cause of Suicide and Race and Ethnicity, United States, 1999-2020 **eTable 3.** Age-Standardized Mortality Rates Among Adolescents by Cause of Suicide and Sex, United States, 1999-2020 **eTable 4.** Annual Percentage Changes in Death Rates Among Adolescents Due to Suicide by Cause, United States, 1999-2020 **eTable 5.** Annual Percentage Changes in Death Rates Among Adolescents Due to Suicide by Race and Ethnicity and Cause, United States, 1999-2020 **eTable 6.** Annual Percentage Changes in Death Rates Among Adolescents Due to Suicide by Sex and Cause, United States, 1999-2020 **eTable 7.** Annual Percentage Changes in Death Rates Among Adolescents Due to Suicide by Age and Cause, United States, 1999-2020 **eTable 8.** Annual Percentage Changes in Death Rates Among Adolescents Due to Suicide Jumping, United States, 1999-2020 **eTable 9.** Annual Percentage Changes in Death Rates Among Adolescents Due to Suicide Drug Poisoning, United States, 1999-2020

Supplement 2.
Data Sharing Statement
