# Supplementary material for: Trends in Adolescent Suicide by Method in the US, 1999-2020
Source: JAMA Netw Open. 2024 Mar 29;7(3):e244427. doi: 10.1001/jamanetworkopen.2024.4427 (PMC10980967; doi:10.1001/jamanetworkopen.2024.4427)
Supplement: Supplement 1. — eTable 1. External Causes of Death ICD-Codes eTable 2. Age-Standardized Mortality Rates Among Adolescents by Cause of Suicide and Race and Ethnicity, United States, 1999-2020 eTable 3. Age-Standardized Mortality Rates Among Adolescents by Cause of Suicide and Sex, United States, 1999-2020 eTable 4. Annual Percentage Changes in Death Rates Among Adolescents Due to Suicide by Cause, United States, 1999-2020 eTable 5. Annual Percentage Changes in Death Rates Among Adolescents Due to Suicide by Race and Ethnicity and Cause, United States, 1999-2020 eTable 6. Annual Percentage Changes in Death Rates Among Adolescents Due to Suicide by Sex and Cause, United States, 1999-2020 eTable 7. Annual Percentage Changes in Death Rates Among Adolescents Due to Suicide by Age and Cause, United States, 1999-2020 eTable 8. Annual Percentage Changes in Death Rates Among Adolescents Due to Suicide Jumping, United States, 1999-2020 eTable 9. Annual Percentage Changes in Death Rates Among Adolescents Due to Suicide Drug Poisoning, United States, 1999-2020 [file jamanetwopen-e244427-s001.pdf]

Supplementary Online Content

Ormiston CK, Lawrence WR, Sulley S, et al. Trends in adolescent suicide by method in the US, 1999-2020. *JAMA Netw Open*. 2024;7(4):e244427. doi:10.1001/jamanetworkopen.2024.4427

eTable 1. External Causes of Death ICD-Codes
eTable 2. Age-Standardized Mortality Rates Among Adolescents by Cause of Suicide and Race and Ethnicity, United States, 1999-2020
eTable 3. Age-Standardized Mortality Rates Among Adolescents by Cause of Suicide and Sex, United States, 1999-2020
eTable 4. Annual Percentage Changes in Death Rates Among Adolescents Due to Suicide by Cause, United States, 1999-2020
eTable 5. Annual Percentage Changes in Death Rates Among Adolescents Due to Suicide by Race and Ethnicity and Cause, United States, 1999-2020
eTable 6. Annual Percentage Changes in Death Rates Among Adolescents Due to Suicide by Sex and Cause, United States, 1999-2020
eTable 7. Annual Percentage Changes in Death Rates Among Adolescents Due to Suicide by Age and Cause, United States, 1999-2020
eTable 8. Annual Percentage Changes in Death Rates Among Adolescents Due to Suicide Jumping, United States, 1999-2020
eTable 9. Annual Percentage Changes in Death Rates Among Adolescents Due to Suicide Drug Poisoning, United States, 1999-2020

This supplementary material has been provided by the authors to give readers additional information about their work.

© 2024 Ormiston CK al. *JAMA Network Open.*

eTable 1. Adolescent Suicide Causes of Death ICD-Codes

Causes/Mechanism	ICD-Codes
Firearm	X72–X74
Poisoning	X60–X69
Drug poisoning	X60-X64
Suffocation/hanging	X70
Jumping	X80
Other	X71, X75, X76, X77, X78, X79, X81, X82, X83, X84

Abbreviations: International Statistical Classification of Diseases and Related Health Problems, Tenth Revision, ICD-10

© 2024 Ormiston CK al. *JAMA Network Open.*

eTable 2. Age-standardized mortality rates among adolescents by cause of suicide and race and ethnicity, United States, 1999-2020

	Age-standardized death rate, per 100,000 population (95% CI)[a]					
			Non-Hispanic individuals			
Suicide Cause	Total Population	Hispanic or Latino individuals	American Indian and Alaska Native[b]	Asian and Pacific Islander	Black	White
Overall	5.1 (5.0, 5.1)	3.7 (3.6, 3.7)	20.3 (19.2, 21.4)	3.5 (3.4, 3.7)	3.4 (3.3, 3.5)	5.9 (5.8, 6.0)
Firearm	2.2 (2.2, 2.2)	1.2 (1.2, 1.3)	6.3 (5.7, 6.9)	0.9 (0.8, 1.0)	1.5 (1.4, 1.5)	2.8 (2.8, 2.9)
Poison	0.3 (0.3, 0.3)	0.2 (0.2, 0.2)	0.8 (0.6, 1.1)	0.3 (0.2, 0.3)	0.2 (0.1, 0.2)	0.4 (0.4, 0.4)
Drug poisoning [C]	0.3 (0.3, 0.3)	0.2 (0.2 0.2)	0.8 (0.6, 1.0)	0.2 (0.2, 0.3)	0.1 (0.1, 0.2)	0.3 (0.3, 0.3)
Hanging/strangulation/suffocation	2.2 (2.2, 2.2)	1.9 (1.9, 2.0)	12.6 (11.7, 13.4)	1.8 (1.7, 1.9)	1.5 (1.5, 1.6)	2.3 (2.3, 2.4)
Other	0.3 (0.3, 0.3)	0.3 (0.2, 0.3)	0.6 (0.5, 0.9)	0.5 (0.5, 0.6)	0.2 (0.2, 0.3)	0.4 (0.3, 0.4)
Jumping [C]	0.1 (0.1, 0.1)	0.1 (0.1, 0.1)	0.2 (0.1, 0.3)	0.3 (0.2, 0.3)	0.1 (0.1, 0.1)	0.1 (0.1, 0.1)

[a] Rates are per 100,000 and age-adjusted to the 2000 US Std Population.
[b] Data for the non-Hispanic American Indian/Alaska Native population are restricted to Indian Health Service Purchased/Referred Care Delivery Area counties.
[C] Includes deaths reported in overall category

© 2024 Ormiston CK al. *JAMA Network Open.*

eTable 3. Age-standardized mortality rates among adolescents by cause of suicide and sex, United States, 1999-2020

	Age-standardized death rate, per 100,000 population (95% CI) [a]	
Suicide Cause	Female	Male
Overall	2.4 (2.3, 2.4)	7.6 (7.5, 7.7)
Firearm	0.6 (0.5, 0.6)	3.8 (3.7, 3.9)
Poison	0.3 (0.3, 0.4)	0.3 (0.3, 0.3)
Drug poisoning [b]	0.3 (0.3, 0.3)	0.2 (0.2, 0.2)
Hanging/strangulation/suffocation	1.3 (1.2, 1.3)	3.0 (3.0, 3.1)
Other	0.2 (0.2, 0.2)	0.5 (0.4, 0.5)
Jumping [b]	0.1 (0.1, 0.1)	0.2 (0.1, 0.2)

[a] Rates are per 100,000 and age-adjusted to the 2000 US Std Population.
[b] Includes deaths reported in overall category

© 2024 Ormiston CK al. *JAMA Network Open.*

eTable 4. Annual percentage changes in death rates among adolescents due to suicide by cause, United States, 1999-2020

Cause of death	AAPC from (1999-2020)	APC Segment 1		APC Segment 2		APC Segment 3	
Firearm	1.0 (0.1, 1.9)	1999-2007	-6.0 (-7.9, -4.2)	2007-2020	5.5 (4.6, 6.5)	NA	NA
Poison	2.7 (1.0, 4.4)	1999-2011	-1.5 (-3.8, 0.8)	2011-2020	8.6 (5.7, 11.7)	NA	NA
Hanging/strangulation/ suffocation	2.4 (0.2, 4.6)	1999-2013	2.8 (1.8, 3.8)	2013-2018	6.4 (0.3, 13.0)	2018-2020	-9.6 (-24.7, 8.6)
Other	2.9 (1.2, 4.6)	1999-2008	-0.7 (-2.8, 1.5)	2008-2015	8.8 (4.8, 13.0)	2015-2020	1.4 (-2.4, 5.4)

Segments were chosen by Joinpoint regression.

Abbreviations: AAPC, Average annual percentage change; APC, Annual percentage change; NA, not applicable

Note: a specific segment assumes that the trend is continuous at the Joinpoint

© 2024 Ormiston CK al. *JAMA Network Open.*

eTable 5. Annual percentage changes in death rates among adolescents due to suicide by race and ethnicity and cause, United States, 1999-2020

Cause of death	AAPC from (1999-2020)	APC Segment 1		APC Segment 2		APC Segment 3		APC Segment 4	
American Indian and Alaska Native									
Firearm	2.5 (0.0, 5.1)	1999-2014	-0.5 (-2.7, 1.7)	2014-2020	10.6 (2.6, 19.3)	NA	NA	NA	NA
Poison	—	—	NA	NA	NA	NA	NA	NA	NA
Hanging/strangulation/suffocation	3.6 (2.2, 5.0)	1999-2020	3.6 (2.2, 5.0)	NA	NA	NA	NA	NA	NA
Other	—	—	NA	NA	NA	NA	NA	NA	NA
Asian and Pacific Islander									
Firearm	1.8 (-0.1, 3.8)	1999-2008	-10.4 (-13.8, -6.8)	2008-2020	12.0 (9.7, 14.5)	NA	NA	NA	NA
Poison	—	—	—	NA	NA	NA	NA	NA	NA
Hanging/strangulation/suffocation	2.3 (-1.2, 5.9)	1999-2013	1.8 (-0.2, 3.8)	2013-2018	14.7 (4.0, 26.5)	2018-2020	-20.2 (-40.0, 6.2)	NA	NA
Other	—	—	—	NA	NA	NA	NA	NA	NA
Black									
Firearm	1.8 (-0.1, 3.9)	1999-2012	-5.2 (-7.2, -3.2)	2012-2020	14.5 (9.7, 19.5)	NA	NA	NA	NA
Poison	—	—	—	NA	NA	NA	NA	NA	NA
Hanging/strangulation/suffocation	4.2 (3.2, 5.2)	1999-2020	4.2 (3.2, 5.2)	NA	NA	NA	NA	NA	NA
Other	—	—	—	NA	NA	NA	NA	NA	NA
Hispanic or Latino									
Firearm	0.8 (-1.0, 2.7)	1999-2011	-5.7 (-7.9, -3.4)	2011-2020	10.2 (6.8, 13.8)	NA	NA	NA	NA
Poison	—	—	—	NA	NA	NA	NA	NA	NA
Hanging/strangulation/suffocation	3.2 (2.4, 4.1)	1999-2020	3.2 (2.4, 4.1)	NA	NA	NA	NA	NA	NA
Other	—	—	—	NA	NA	NA	NA	NA	NA

© 2024 Ormiston CK al. *JAMA Network Open.*

White									
Firearm	1.1 (-0.1, 2.3)	1999-2007	-5.2 (-6.8, -3.6)	2007-2017	7.1 (5.7, 8.6)	2017-2020	-1.3 (-7.6, 5.4)	NA	NA
Poison	2.0 (0.3, 3.8)	1999-2010	-2.3 (-4.8, 0.2)	2010-2020	7.1 (4.2, 10.1)	NA	NA	NA	NA
Hanging/strangulation/suffocation	2.4 (-0.7, 5.6)	1999-2004	6.2 (1.5, 11.1)	2004-2007	-3.2 (-20.1, 17.3)	2007-2018	5.4 (4.0, 6.8)	2018-2020	-13.1 (-27.0, 3.4)
Other	3.5 (2.6, 4.4)	1999-2020	3.5 (2.6, 4.4)	NA	NA	NA	NA	NA	NA

Segments were chosen by Joinpoint regression.
Abbreviations: AAPC, Average annual percentage change; APC, Annual percentage change; NA, not applicable
Note: a specific segment assumes that the trend is continuous at the joinpoint
“—” = not calculable, including suppressed data for counts <10 for any years
Data for the non-Hispanic American Indian/Alaska Native Population are restricted to Indian Health Service Purchased/Referred Care delivery Area counties.

© 2024 Ormiston CK al. *JAMA Network Open.*

eTable 6. Annual percentage changes in death rates among adolescents due to suicide by sex and cause, United States, 1999-2020

Cause of death	AAPC from (1999-2020)	APC Segment 1		APC Segment 2		APC Segment 3		APC Segment 4	
Female									
Firearm	1.5 (-0.1, 3.1)	1999-2007	-8.0 (-11.3, -4.4)	2007-2020	7.8 (6.0, 9.5)	NA	NA	NA	NA
Poison	4.5 (2.3, 6.7)	1999-2011	-1.2 (-4.0, 1.7)	2011-2020	12.6 (8.5, 16.7)	NA	NA	NA	NA
Hanging/ strangulation/suffocation	5.9 (5.0, 6.8)	1999-2020	5.9 (5.0, 6.8)	NA	NA	NA	NA	NA	NA
Other	3.1 (0.3, 5.9)	1999-2008	-2.4 (-6.3, 1.5)	2008-2015	14.6 (8.3, 21.3)	2015-2020	-2.0 (-8.1, 4.6)	NA	NA
Male									
Firearm	0.9 (0.0, 1.9)	1999-2007	-5.8 (-7.7, -3.8)	2007-2020	5.3 (4.3, 6.3)	NA	NA	NA	NA
Poison	1.0 (-0.1, 2.1)	1999-2020	1.0 (-0.1, 2.1)	NA	NA	NA	NA	NA	NA
Hanging/ strangulation/suffocation	1.4 (-1.2, 4.1)	1999-2015	1.7 (1.0, 2.5)	2015-2018	10.5 (-6.0, 29.9)	2018-2020	-13.0 (-26.4, 2.8)	NA	NA
Other	3.2 (2.3, 4.1)	1999-2020	3.2 (2.3, 4.1)	NA	NA	NA	NA	NA	NA

Segments were chosen by Joinpoint regression.

Abbreviations: AAPC, Average annual percentage change; APC, Annual percentage change; NA, not applicable

Note: a specific segment assumes that the trend is continuous at the Joinpoint

© 2024 Ormiston CK al. *JAMA Network Open.*

eTable 7. Annual percentage changes in death rates among adolescents due to suicide by age and cause, United States, 1999-2020

Cause of death	AAPC from (1999-2020)	APC Segment 1		APC Segment 2		APC Segment 3		APC Segment 4	
Ages 10-14									
Firearm	3.1 (0.3, 6.0)	1999-2008	-7.8 (-10.9, -4.6)	2008-2014	20.2 (10.8, 30.3)	2014-2020	4.6 (0.2, 9.3)	NA	NA
Poison	7.6 (4.8, 10.4)	1999-2020	7.6 (4.8, 10.4)	NA	NA	NA	NA	NA	NA
Hanging/strangulation/suffocation	3.8 (1.9, 5.8)	1999-2008	-0.7 (-4.5, 3.3)	2008-2020	7.3 (5.2, 9.4)	NA	NA	NA	NA
Other	–	–	NA	NA	NA	NA	NA	NA	NA
Ages 15-19									
Firearm	0.3 (-2.1, 2.8)	1999-2007	-5.3 (-7.0, -3.6)	2007-2014	2.9 (-0.1, 5.9)	2014-2017	12.3 (-4.0, 31.4)	2017-2020	-1.5 (-8.4, 5.9)
Poison	2.2 (0.7, 3.8)	1999-2011	-1.4 (-3.3, 0.6)	2011-2020	7.3 (4.5, 10.1)	NA	NA	NA	NA
Hanging/strangulation/suffocation	2.1 (0.5, 3.8)	1999-2018	3.5 (2.9, 4.0)	2018-2020	-9.7 (-23.9, 7.1)	NA	NA	NA	NA
Other	3.4 (1.6, 5.2)	1999-2007	0.2 (-3.9, 4.4)	2007-2020	5.4 (3.8, 7.2)	NA	NA	NA	NA

Segments were chosen by Joinpoint regression.
Abbreviations: AAPC, Average annual percentage change; APC, Annual percentage change; NA, not applicable
"–" = not calculable.
Note: a specific segment assumes that the trend is continuous at the joinpoint

© 2024 Ormiston CK al. *JAMA Network Open.*

eTable 8. Annual percentage changes in death rates among adolescents due to suicide jumping, United States, 1999-2020

	AAPC from (1999-2020)	APC Segment 1		APC Segment 2		APC Segment 3		APC Segment 4	
Jumping	3.3 (1.9, 4.8)	1999-2008	-3.7 (-6.5, -0.8)	2008-2020	8.9 (7.4, 10.5)	NA	NA	NA	NA
Sex									.
Female	–	–	–	–	–	NA	NA	NA	NA
Male	3.2 (1.0, 5.4)	1999-2008	-3.0 (-7.1, 1.4)	2008-2020	8.1 (5.7, 10.5)	NA	NA	NA	NA
Age Group									
10-14	–	–	NA	NA	NA	NA	NA	NA	NA
15-19	3.3 (1.6, 5.0)	1999-2008	-3.6 (-6.9, -0.3)	2008-2020	8.8 (6.9, 10.7)	NA	NA	NA	NA
Race and Ethnicity									
American Indian and Alaska Native	–	–	NA	NA	NA	NA	NA	NA	NA
Asian and Pacific Islander	–	–	–	NA	NA	NA	NA	NA	NA
Black	–	–	–	–	–	NA	NA	NA	NA
Hispanic or Latino	–	–	–	NA	NA	NA	NA	NA	NA
White	2.9 (-0.2, 6.0)	1999-2008	-2.5 (-8.2, 3.5)	2008-2020	7.1 (3.4, 10.9)	NA	NA	NA	NA

Segments were chosen by Joinpoint regression.
Abbreviations: AAPC, Average annual percentage change; APC, Annual percentage change; NA, not applicable
Note: a specific segment assumes that the trend is continuous at the joinpoint
"–" = not calculable, including suppressed data for counts <10 for any years

© 2024 Ormiston CK al. *JAMA Network Open.*

eTable 9. Annual percentage changes in death rates among adolescents due to suicide drug poisoning, United States, 1999-2020

	AAPC from (1999-2020)	APC Segment 1		APC Segment 2		APC Segment 3		APC Segment 4	
Drug poisoning	3.5 (1.8, 5.1)	1999-2012	-0.0 (-1.9, 1.8)	2012-2020	9.4 (5.8, 13.1)	NA	NA	NA	NA
Sex									
Female	4.8 (2.5, 7.1)	1999-2011	-0.7 (-3.7, 2.3)	2011-2020	12.5 (8.5, 16.8)	NA	NA	NA	NA
Male	1.4 (0.2, 2.6)	1999-2020	1.4 (0.2, 2.6)	NA	NA	NA	NA	NA	NA
Age Group									
10-14	–	–	–	NA	NA	NA	NA	NA	NA
15-19	3.0 (1.5, 4.5)	1999-2012	0.3 (-1.3, 2.0)	2012-2020	7.5 (4.3, 10.8)	NA	NA	NA	NA
Race and Ethnicity									
American Indian and Alaska Native	–	–	NA	NA	NA	NA	NA	NA	NA
Asian and Pacific Islander	–	–	–	NA	NA	NA	NA	NA	NA
Black	–	–	–	NA	NA	NA	NA	NA	NA
Hispanic or Latino	–	–	NA	NA	NA	NA	NA	NA	NA
White	2.8 (1.0, 4.7)	1999-2010	-0.5 (-3.2, 2.2)	2010-2020	6.6 (3.8, 9.5)	NA	NA	NA	NA

Segments were chosen by Joinpoint regression.
Abbreviations: AAPC, Average annual percentage change; APC, Annual percentage change; NA, not applicable
Note: a specific segment assumes that the trend is continuous at the joinpoint
"–" = not calculable, including suppressed data for counts <10 for any years

© 2024 Ormiston CK al. *JAMA Network Open.*
