# Supplementary material for: Trends in Adolescent Suicide by Method in the US, 1999-2020
Source: JAMA Netw Open. 2024 Mar 29;7(3):e244427. doi: 10.1001/jamanetworkopen.2024.4427 (PMC10980967; doi:10.1001/jamanetworkopen.2024.4427)
Supplement: Supplement 2. — Data Sharing Statement [file jamanetwopen-e244427-s002.pdf]

Data Sharing Statement

Ormiston. Trends in Adolescent Suicide by Method in the US, 1999-2020. *JAMA Netw Open*. Published April 01, 2024. doi:10.1001/jamanetworkopen.2024.4427

Data
Data available: No
